# Longitudinal neuropsychological trajectories in idiopathic normal pressure hydrocephalus: a population–based study

**DOI:** 10.1186/s12877-023-03747-y

**Published:** 2023-01-16

**Authors:** Otto Lilja-Lund, Martin Maripuu, Karin Kockum, Johanna Andersson, Anna Lindam, Lars Nyberg, Katarina Laurell

**Affiliations:** 1 Department of Clinical Sciences, Neuroscience, Umeå University, Umeå, Sweden; 2 Department of Clinical Sciences, Psychiatry, Umeå University, Umeå, Sweden; 3 Department of Public Health and Clinical Medicine, Unit of Research, Education and Development Östersund Hospital, Umeå University, Umeå, Sweden; 4 Department of Radiation Sciences, Radiology, Umeå University, Umeå, Sweden; 5 Department of Integrative Medical Biology, Umeå University, Umeå, Sweden; 6 Umeå Center for Functional Brain Imaging, Umeå University, Umeå, Sweden; 7 Center for Lifespan Changes in Brain and Cognition, University of Oslo, Oslo, Norway; 8 Department of Medical Sciences, Neurology, Uppsala University, Uppsala, Sweden

**Keywords:** Idiopathic normal pressure hydrocephalus, Ageing, Older adults, Cognition, Neuropsychology, Life-span, Cognitive development, Population-based

## Abstract

**Background:**

Idiopathic normal pressure hydrocephalus (iNPH) is a progressive syndrome affecting gait, incontinence, and cognition in a significant number of older adults. Still, prospective studies on early development of symptoms are scarce.

**Aim:**

To investigate how neuropsychological functions develop before and in already diagnosed iNPH over a two-year period in a population-based material.

**Method:**

A sample of 104 participants (median [IQR] 75 [72–80] years old) from the general population underwent CT-imaging and clinical assessment at baseline and follow-up. We used the iNPH symptom scale covering four domains (Neuropsychology, Gait, Balance, Incontinence) and additional tests of executive functions. Morphological signs were rated with the iNPH Radscale. Non-parametric statistics with Bonferroni corrections and a significance-level of *p* < 0.05 were used.

**Results:**

Median (IQR) time to follow-up was 25 (23–26) months. Effect size (ES) for individuals who developed iNPH (*n* = 8) showed a large (ES *r* = -0.55) decline in the Gait domain and on the Radscale (ES *r* = -0.60), with a medium deterioration in declarative memory (ES *r* = -0.37). Those having iNPH at baseline (*n* = 12) performed worse on one executive sub-function i.e., shifting (*p* = 0.045).

**Conclusion:**

Besides deterioration in gait and radiology, our results suggest that a neuropsychological trajectory for those developing iNPH includes a reduction in declarative memory. Executive dysfunction was limited to those already having iNPH at baseline. These findings could suggest that memory impairments are included in the early development of iNPH.

## Background

Idiopathic normal pressure hydrocephalus (iNPH) is a progressive syndrome with a negative effect on the “triad” of gait, cognition, and incontinence [1, 2]. There are distinct radiological features on brain imaging such as an Evans’ index ≥ 0.3 [2, 3], wide temporal horns [4, 5], and a narrow callosal angle [6]. Typically, adults aged 65 years or older are affected by iNPH with an estimated prevalence of 1.4–3.7% that increases with age [7, 8]. Clearance of excess cerebrospinal fluid (CSF) with shunting is an effective treatment and waiting-time to surgery can negatively impact the outcome; hence, timely interventions are important [9, 10].

Gait disturbance is the most frequent symptom in iNPH present in 90–100% of diagnosed cases followed by worsened cognition (56–98%), and incontinence (60–92%) [2, 11]. Although gait disturbances are hallmark signs of iNPH, upper motor dysfunction is a part of the syndrome as well [12, 13]. One prominent cognitive dysfunction in iNPH is poor executive functioning [14, 15]. Executive functioning can be divided in three separate, but related domains; shifting, updating, and inhibition, with a prominent frontal-lobe contribution [16, 17]. Another salient form of cognitive disturbance is decreased verbal memory [18–20]. Verbal memory tests such as the Rey Auditory Verbal Learning Test (RAVLT) taps into the ability to learn, store, and retrieve verbal information thus reflecting declarative memory [21, 22].

Few studies have focused on the developmental trajectory of iNPH [23]. Additionally, not all but most studies are retrospective with a focus on radiological signs of iNPH before symptom onset, referred to as AVIM (asymptomatic ventriculomegaly with features of iNPH on MRI) [24–27]. One of these studies found that those who developed triad symptoms e.g., gait disturbances or incontinence, did not worsen on the neuropsychological tests after one year [27]. In one longitudinal single-case-study, the patient was followed with annual radiological examinations for 16 years [28]. The patient had signs of gait disturbances seven years prior to diagnosis, incontinence six years prior, and cognitive decline one year prior to diagnosis [28].

Prospective studies with a focus on the development of neuropsychological functions iNPH are lacking, preferably including sensitive methods to reveal subtle pre-diagnostic signs. More extensive neuropsychological testing can potentially delineate the progress better than cognitive screening with low sensitivity, and a broader neuropsychological test battery might differentiate between a general cognitive decline versus impairment in specific domains.

Also, the extent of changes may vary depending on diagnosis at baseline and follow-up. One might expect small changes for those who remain unlikely to have iNPH, but larger changes if one deteriorates to having iNPH at follow-up. The change in function for those already having iNPH at baseline is more uncertain, but one possibility is that they have reached a “floor” in their decline and therefor does not change as much between baseline and follow-up. Thus, the main aim of the study was to assess changes in neuropsychological functions related to iNPH over 2-years in older community dwelling adults.

## Method

### Study participants

**Fig. 1:**
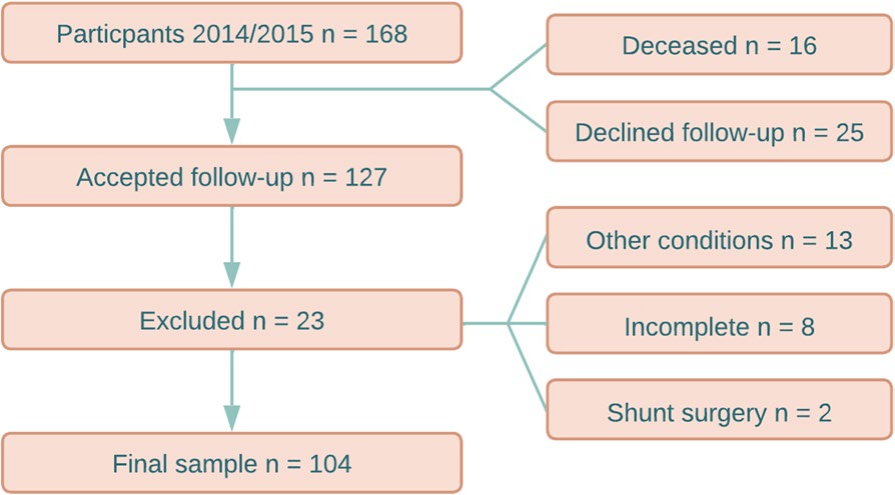
Selection flow-chart over participants. In 2014/15 168 older adults participated in the baseline study. Thirteen of the 23 participants lost at follow-up were excluded based on conditions severely affecting gait and/or cognition (Alzheimer’s disease, hip-surgery, cancer, visual impairment, spinal stenosis, and secondary hydrocephalus). Three declined neurological examination, three had incomplete neuropsychological tests, and two declined imaging. Two participants underwent shunt surgery after 2014/15

The participants were recruited from an epidemiological study on iNPH among inhabitants 65 years or older in a northern region of Sweden [4, 7, 29–33]. A detailed description of the selection is available in Andersson et al. [7]. Out of the original 168 participants at baseline in 2014/15, 127 participated at follow-up in 2016/17 of which 104 were included in the present study. Those considered as dropouts (*n* = 51) included the deceased after baseline (*n* = 16), those who declined follow-up (*n* = 25), incomplete assessment at follow-up (*n* = 8), and those having had shunt-surgery after baseline (*n* = 2). Comparisons between dropouts and participants at follow-up are presented in Table 1. The selection flow-chart is presented in Fig. 1.

**Table 1:** Descriptive statistics and comparisons between dropouts and participants in the longitudinal study

	Dropouts	Longitudinal	χ^2^	*p*
n (female)	51 (55%)	104 (55%)	0.01	0.991
Unlikely / iNPH	65% / 35%	87% / 13%	9.96	**0.002**
	Md (IQR)	Md (IQR)	U	
Age yrs	75 (70–83)	73 (70–78)	2125	**0.044**
iNPH Radscale	3 (1–5)	2 (1–3)	2006	**0.012**
iNPH Total symptom	69 (51–86)	87 (75–94)	3896	**< 0.001**

Significant values of *p* in bold

*Md* Median, *IQR* Interquartile range

### Neuropsychological testing

The participants general cognition was screened with the MMSE [34]. Declarative memory was assessed with the Rey Auditory Verbal Learning Test (RAVLT) [35, 36]. Executive functions were assessed with the Stroop test, Trail-making-test B (TMT B), and digit-span [35, 37]. Upper motor function and psychomotor speed were assessed with the TMT A, and the Grooved Pegboard Test (GPT; Lafayette Instrument Co., Lafayette, IN, USA) [35, 37]. Symptom severity was graded with the iNPH symptom scale covering four domains: Neuropsychology (RAVLT, Stroop, GPT), Balance (rating of a balance task), Continence (symptom rating), and Gait (rating of gait and timed 10-m walking) [35]. Scores on the iNPH symptom scale ranges from 0–100, with low scores representing worse performance.

### Method of diagnosing

The Japanese guidelines 2^nd^ edition was used to diagnose either Unlikely or Possible iNPH based on clinical symptoms and imaging support [29, 38]. The modified Rankin Scale (mRS) was used for rating functional capacity [39]. On the baseline radiological examination, the participants underwent a non-contrast enhanced computed tomography (CT) of the brain (Philips Ingenuity 2013, 128 channels). Non-contrast CT was repeated at follow-up (GE MD Optima CT540), generating the same slice thickness and reconstructions as baseline. A detailed description of the imaging-protocol has previously been described in Kockum et al. [30]. Radiological features were assessed by a radiologist and scored using the iNPH Radscale [30]. The scores on the iNPH Radscale varies from 0–12 with zero representing the absence of features associated with iNPH.

### Statistical analyses

Assumption of normal distribution was investigated with Q–Q plots, histograms, and tested with the Shapiro–Wilk test. Non-parametric analyses were used based on distribution and type of variable. Chi^2^ or Fisher’s exact test were used to examine categorical variables. The Mann–Whitney U, Wilcoxon signed rank, and Kruskal–Wallis tests were used for group-wise comparisons. We used Bonferroni corrected *post-hoc* tests. The non-parametric effect size (ES) *r* was calculated and the magnitude of change was evaluated according to Cohen’s criteria (0.5 = large, 0.3 = medium, 0.1 = small) [40]. We applied two-tailed tests with the level for statistically significant results at *p* < 0.05. Statistical analyses were conducted using IBM SPSS Statistics 27 (IBM Corp., Armonk, NY, USA).

## Results

We compared if those with diagnosed iNPH or Unlikely iNPH at follow-up differed on sex, age, functional capacity, or education. Those with iNPH (*n* = 20) had more morphological features and symptoms of iNPH, were older, and had a higher degree of disability compared to those with Unlikely iNPH (*n* = 84) at follow-up. The two groups did not differ in level education or distribution of sex. Detailed results are presented in Table 2.

**Table 2:** Descriptive statistics of participants diagnosed at follow-up

	iNPH	Unlikely	χ^2^	*p*
n (female%)	20 (45%)	84 (57%)	0.96	0.327
	Md (IQR)	Md (IQR)	U	
Age yrs	80 (75–82.5)	74 (72–78)	493	**0.004**
Education yrs	9 (7–12.5)	9.5 (7–12)	773	0.582
MMSE	27 (26–28)	27 (26–28)	804	0.831
mRS	2 (1–2)	1 (0–1)	505	**0.008**
iNPH Radscale	4 (3–5)	2 (1–3)	305	**< 0.001**
iNPH Total symptom	74 (67–81)	90 (81–95)	301	**< 0.001**

Significant values of *p* in bold

*Md* Median, *IQR* Inter quartile range, *MMSE* Mini-Mental State Examination, *mRS* Modified Rankin Scale

When analyzing changes in diagnosis between baseline and follow-up we found two participants that went from iNPH to Unlikely, eight from Unlikely to iNPH, and 94 who remained unchanged. Three groups were formed based on diagnostic status at baseline and follow-up; those who remained iNPH (*n* = 12), developed iNPH (*n* = 8), or remained Unlikely iNPH (*n* = 82). There were no differences in education or distribution of sex between the groups.

There was a tendency for those who developed iNPH being older than the those who remained as Unlikely iNPH (*p* = 0.074). Those who remained as iNPH had inferior results compared to those who remained as Unlikely at follow-up on the mRS; iNPH Radscale; Total-, Neuropsychology-, Gait-, and Continence iNPH score; GPT; and RAVLT Immediate at baseline. Those who remained as iNPH also had worse results on RAVLT Immediate compared to those who developed iNPH. Baseline results for the three groups are presented in Table 3.

**Table 3:** Baseline descriptive statistics and Kruskal–Wallis tests for groups based on diagnosis at baseline and follow-up

	Remained iNPH	Developed iNPH	Remained Unlikely	χ^2^ (2)	*p*
n (female%)	12 (33%)	8 (63%)	82 (57%)	2.63	0.266
	Md (IQR)	Md (IQR)	Md (IQR)	H (2)	
Age yrs	78 (72–83)	79 (73–81)	72 (70–76)	8.33	**0.016**
Education yrs	9 (7–10)	11 (8–17)	10 (7–12)	2.13	0.345
MMSE	29 (26–29)	29 (27–30)	28 (27–30)	0.16	0.923
mRS	2 (1–2) **a**	1 (1–2)	1 (0–1)	10.51	**0.005**
iNPH Radscale	5 (3–6) **a**	2 (1–3)	2 (1–3)	20.84	**< 0.001**
iNPH Total symptom	75 (67–81) **a**	86 (74–88)	91 (80–95)	13.40	**0.001**
*iNPH Domain*					
Neuropsychology	71 (59–79) **a**	84 (63–97)	83 (78–93)	10.80	**0.005**
Gait	77 (71–89) **a**	94 (88–97)	95 (86–100)	8.57	**0.014**
Balance	83 (67–83)	83 (83–100)	83 (83–100)	5.37	0.068
Continence	60 (40–80) **a**	60 (60–95)	100 (60–100)	9.54	**0.008**
*Memory*					
RAVLT Immediate	26 (19–30) **a**	34 (28–42) **b**	34 (27–38)	9.35	**0.009**
*Executive function*					
Stroop Color	81 (63–86)	65 (62–87)	69 (61–81)	2.09	0.352
Stroop Interference	153 (133–227)	135 (118–219)	139 (115–159)	3.47	0.176
*Motor function*					
10-m time sec	10 (9–10)	9 (8–9)	9 (7–10)	5.20	0.074
10-m steps	17 (16–18)	16 (14–17)	15 (14–17)	4.91	0.086
GPT sec	94 (86–115) **a**	91 (71–115)	80 (68–91)	8.21	**0.016**

Significant values of p in bold

*Md* Median, *IQR* Interquartile range, *MMSE* Mini-Mental State Examination, *mRS* Modified Rankin Scale, *TUG* Timed-Up-Go test, *GPT* Grooved Pegboard Test, *RAVLT* Rey Auditory Verbal Learning Test

Significant post-hoc: **a** =Remained iNPH vs. Remained Unlikely; **b** = Remained iNPH vs. Developed iNPH; **c** = Developed iNPH vs. Remained Unlikely

A similar pattern was found at follow-up, with some exceptions. Those who developed iNPH had worse total Radscale score compared to those who remained as Unlikely at follow-up. In addition, those who remained as iNPH or Unlikely differed on the Balance domain, and all tests of motor function at follow-up. Those who developed iNPH did no longer perform better on the RAVLT Immediate memory test compared to those who remained iNPH. Of the added neuropsychological tests at follow-up those who remained as iNPH performed worse compared to those who remained as Unlikely on the RAVLT Delayed and the TMT B. Performance on the forward digit-span was almost identical, whereas backwards digit-span differed significantly on the Kruskal–Wallis, but not on the adjusted *post-hoc*. Results from follow-up are presented in Table 4.

**Table 4:** Follow-up descriptive statistics and Kruskal–Wallis tests for groups based on diagnosis at baseline and follow-up

	Remained iNPH	Developed iNPH	Remained Unlikely		
	*n* = 12	*n* = 8	*n* = 82		
	Md (IQR)	Md (IQR)	Md (IQR)	H (2)	*p*
MMSE	27 (25–28)	27 (26–29)	27 (26–28)	0.27	0.874
mRS	2 (1–2) **a**	1 (0–2)	1 (0–1)	9.38	**0.009**
iNPH Radscale	5 (3–6) **a**	4 (2–5) **c**	2 (1–3)	21.02	**< 0.001**
iNPH Total symptom	71 (67–76) **a**	80 (63–87)	89 (80–95)	20.72	**< 0.001**
*iNPH Domain*					
Neuropsychology	71 (58–84) **a**	84 (59–97)	85 (73–93)	6.86	**0.032**
Gait	73 (59–81) **a**	85 (65–92)	95 (85–100)	17.83	**< 0.001**
Balance	75 (67–83) **a**	83 (83–96)	83 (83–100)	11.40	**0.003**
Continence	60 (40–80) **a**	80 (45–80)	100 (75–100)	11.28	**0.004**
*Memory*					
RAVLT Immediate	27 (20–29) **a**	31 (23–41)	34 (27–38)	6.71	**0.035**
RAVLT Delayed	3 (1–5) **a**	5 (3–7)	5 (4–8)	6.52	**0.038**
*Executive function*					
TMT B	140 (83–204) **a**	72 (56–86)	79 (60–130)	6.63	**0.036**
Max DS forward	6 (4–6)	6 (5–7)	6 (5–6)	1.97	0.373
Max DS backward	3 (2–4)	4 (4–4)	4 (3–5)	6.14	**0.047**
Stroop Color	76 (67–90)	66 (62–86)	68 (60–77)	3.83	0.148
Stroop Interference	145 (134–196)	131 (113–213)	132 (115–166)	3.45	0.178
*Motor function*					
10-m time sec	10 (10–11) **a**	10 (9–12)	9 (8–10)	14.80	**< 0.001**
10-m steps	17 (16–20) **a**	15 (15–17)	15 (14–16)	14.03	**< 0.001**
GPT sec	101 (84–132) **a**	97 (70–119)	86 (72–100)	6.22	**0.045**
TMT A	35 (29–43)	28 (23–35)	29 (23–36)	3.92	0.141

Significant values of p in bold

*Md* Median, *IQR* Interquartile range, *MMSE* Mini-Mental State Examination, *mRS* Modified Rankin Scale, *TUG* Timed-Up-Go test, *GPT* Grooved Pegboard Test, *TMT* Trail Making Test, *RAVLT* Rey Auditory Verbal Learning Test, *DS* Digit-span

Significant post-hoc: **a** = Remained iNPH vs. Remained Unlikely; **b** = Remained iNPH vs. Developed iNPH; **c** = Developed iNPH vs. Remained Unlikely

When we compared baseline results with follow-up results, we found that all three groups had worsened on the MMSE, with a medium to large effect size according to Cohen’s guidelines [40]. Both those who developed iNPH and remained as Unlikely worsened on the Radscale, with a large effect for the prior and small for the latter. The Total symptom score, Gait domain, and 10-m walking worsened for those who developed iNPH, with a large effect on all variables. The Neuropsychology domain and RAVLT Immediate worsened with a medium effect for those who developed iNPH. Those who remained as iNPH also worsened on the Gait domain and 10-m walking time with a medium and large effect respectively. Those who remained as Unlikely improved on number of steps but worsened on the GPT with a medium effect on measures of motor function. See Table 5.

**Table 5:** Wilcoxon signed rank test and effect size comparing change over time for groups based on diagnosis at baseline and follow-up

	Remained iNPH *n* = 12			Developed iNPH *n* = 8			Remained Unlikely *n* = 82		
	z	*p*	ES *r*	z	*p*	ES *r*	z	*p*	ES *r*
MMSE	-2.57	**0.010**	-0.52	-2.43	**0.016**	-0.61	-5.43	**< 0.001**	-0.42
mRS	-1.67	0.188	-0.34	-1.73	0.250	-0.43	-1.25	0.243	-0.10
iNPH Radscale	-0.79	0.410	-0.16	-2.41	**0.016**	-0.60	-2.88	**0.004**	-0.22
iNPH Total symptom	-1.02	0.170	-0.21	-2.10	**0.039**	-0.53	-0.59	0.560	-0.05
*iNPH Domain*									
Neuropsychology	-0.97	0.361	-0.20	-1.41	0.312	-0.35	-0.59	0.561	-0.05
Gait	-2.30	**0.020**	-0.47	-2.20	**0.031**	-0.55	-0.27	0.794	-0.02
Balance	-0.54	0.781	-0.11	-0.76	0.625	-0.19	-0.85	0.423	-0.07
Continence	-0.27	1.000	-0.06	-0.45	1.000	-0.11	-0.16	0.990	-0.01
*Memory*									
RAVLT Immediate	-0.20	0.869	-0.04	-1.48	0.172	-0.37	-0.02	0.983	0.00
*Executive function*									
Stroop Color	-0.55	0.609	-0.11	-0.84	0.453	-0.21	-1.25	0.213	-0.10
Stroop Interference	-0.94	0.380	-0.19	-2.39	**0.016**	-0.60	-0.49	0.626	-0.04
*Motor function*									
10-m time sec	-2.43	**0.012**	-0.50	-2.52	**0.008**	-0.63	-0.30	0.764	-0.02
10-m steps	-1.56	0.132	-0.32	-2.39	**0.016**	-0.60	-3.48	**< 0.001**	-0.27
GPT	-0.82	0.438	-0.17	-0.95	0.406	-0.24	-3.57	**< 0.001**	-0.28

Descriptive statistics are available in Table 3 and 4. Significant values of p in bold

*ES* Effect size, *MMSE* Mini-Mental State Examination, *mRS* Modified Rankin Scale, *TUG* Timed-Up-Go test, *GPT* Grooved Pegboard Test, *RAVLT* Rey Auditory Verbal Learning Test

## Discussion

We investigated how the neuropsychological traits of iNPH developed over time in this prospective, population-based study. We found that those who developed iNPH during the study period had intact declarative memory at baseline and that their morphological features on imaging and gait performance worsened the most. Those who already had iNPH  at baseline performed worse on one executive sub-function, shifting, but not updating or inhibition compared to the other two groups. Furthermore, worsened gait was exclusive to iNPH in our sample.

We found that those who developed iNPH had better declarative memory at baseline compared to those who already had iNPH from start, but this difference diminished at follow-up. At follow-up, those who remained as iNPH and those who developed iNPH did not differ anymore in declarative memory. The change was of a medium effect size for those who developed iNPH. Our results showing worsened declarative memory is in line with other studies highlighting poor declarative memory in iNPH [19, 41]. One example of memory impairment in iNPH is having to stop walking while recalling a recent event [32]. It is conceivable that the reduced memory performance in iNPH is related to the widening of the temporal horns (TH). The TH are the ventricular space adjacent the hippocampus, and wide TH are associated with iNPH [4, 5, 30]. Other studies have found reduced hippocampal cerebral blood-flow in iNPH compared to healthy individuals [42, 43]. Still, better memory but worse executive functions are reported in iNPH compared to Alzheimer’s disease (AD) [44, 45]. To further complicate matters, AD and iNPH can co-exist and have overlapping symptomatology [46–48]. Nevertheless, our findings points to the relevance of considering deteriorating memory functions in developing iNPH, even if they are less pronounced than in AD [20, 45].

We also found variations in executive functions. Those having had iNPH since baseline performed worse on the task involving shifting (TMT B). Performance on the TMT A did not differ between groups, hence difficulties on TMT B are likely attributed to the added executive component and less affected by potential differences in visual scanning and eye-hand-coordination [37]. Unfortunately, we only had access to TMT B at follow-up. However, another study found that TMT B scores were worse for individuals who later were classified as having iNPH [49]. We cannot infer if this would have been the case in our study, but our results at follow-up coincides with other studies showing difficulties with shifting in iNPH [20, 49].

Concerning tasks assessing updating, we found that forward digit-span was very similar for all groups. A recent review on cognition in iNPH presented inconclusive results with regards to impaired updating [20]. According to our results there was only a tendency for difficulties with updating on backwards digit-span, hence problems with updating may be present, but not as prominent as impaired shifting.

Furthermore, performance on the inhibition task (Stroop Interference) did not differ between the groups in our sample. In a previous study by our group on the association between morphological features and neuropsychological signs of iNPH we found that a sharper callosal angle was associated with worse performance on the Stroop Interference test [4]. The participants in the study from 2020 included both dropouts and those who chose to participate at follow-up in this current study, see Fig. 1. The dropouts were older, had iNPH more frequently, and worse Radscale and Total iNPH symptom scores. Hence, it is possible that results on the Stroop Interference test in our current study was affected by a selection-bias in favor of more fit participants. Other studies have found that results on the Stroop Interference test were worse in iNPH [50–52]. Hence, difficulties relating to inhibition in iNPH is somewhat inconclusive, but problems with inhibition was not present in this study.

The neural organization behind the multi-factorial model for executive functions is not completely established, but the basal ganglia and the frontal cortex are proposed to be involved [53, 54]. Frank et al. [53] draws a parallel between the way the frontal cortex and basal ganglia interacts in motor control to how the interaction plays out in working memory. They argue that the basal ganglia contributes to task-selection important for shifting, and the frontal cortex to maintaining or updating [53]. Inhibition is a third factor in the model, and the cingulate cortex is associated with performance on the Stroop Interference task, tapping into inhibition [55]. Interestingly, these are all frontosubcortical regions previously associated with iNPH [56–59]. In summary, we found that shifting, not inhibition or updating, was related to iNPH in our sample.

INPH can manifest itself with a complex cognitive profile, ranging from a global deficit most typical in older age, a frontosubcortical type, lower results on a single neuropsychological task only, or even with intact cognition [11, 45, 60, 61]. Our participants also presented a variation in cognitive deficiency and focusing on one specific cognitive aspect or test might not be the best practice. Thus, the use of broader, sensitive test batteries can be helpful in capturing this heterogeneous manifestation [60, 62]. Also, the use of insensitive cognitive tests could potentially delay or mislead the clinician with false negative results. For instance, we could not identify iNPH in our sample from the general population with the MMSE, but more sensitive neuropsychological tests could. Furthermore, even though the MMSE score worsened over time, this was true for all groups and likely attributed to a general effect of ageing [21].

Gait is one of the most prominent clinical features in iNPH and the decrease in Gait score for those who developed iNPH was in line with the shift in diagnosis [2]. The Gait score worsened for those already having iNPH at baseline as well, and reduced gait function over time might decrease the functional capacity further [63]. Hence, preventing further worsening in gait would be beneficial, and shunt-surgery can be helpful in this [2, 63–65]. Those who remained as Unlikely improved on the 10-m walking at follow-up, contrary to those diagnosed with iNPH. Still, results on the GPT had worsened for those who remained as Unlikely at follow-up, meaning that some motor functions might deteriorate with age, i.e., manual dexterity [66]. Interestingly, worsened gait was specific to iNPH in our study.

Two participants went from iNPH at baseline to Unlikely iNPH at follow-up. The reason for this was a small improvement in the EI ending up below the diagnostic threshold for iNPH [38]. This highlights a challenge with a strict diagnostic criterion. It is necessary to reach consensus across studies, but in a clinical setting one might be more interested in monitoring patients at risk of developing iNPH without a strict cut-off.

A strength in our prospective study was that the participants were recruited from the general population, as most studies on iNPH are from selected material already enrolled in clinics. Our study also has some limitations. The dropouts were older and had iNPH more frequently. This meant that there was a risk of selection bias in favor of more healthy participants, as well as loss of statistical power. This could potentially have led to underestimating the degree of difficulties. Also, capturing enough participants not having symptoms at the start of the study later developing possible iNPH at follow-up was difficult to estimate. Nevertheless, a considerable number proved to develop possible iNPH after two years. Further analysis of incidence, as well as longitudinal radiological changes, are topics for planned papers.

Two different scanners were used but potential differences were controlled by an experienced neuroradiologist and longitudinal comparison of images were deemed to be in order [67]. The protocol was similar between the two scanners generating identical slice thickness and reformats to minimize any technical differences between the scans.

Some participants already fulfilled an iNPH diagnosis at baseline and we do not know how or when their symptoms developed before the start of our study. Future studies should follow the participants over a longer period and preferably include more participants.

## Conclusion

We found that developing iNPH was associated with deterioration in gait and worsened declarative memory. Only those already having iNPH at baseline showed signs of executive dysfunction. In summary, our results could indicate a neuropsychological trajectory for developing iNPH with worsened gait and reduced declarative memory and possibly later onset of executive dysfunction. Memory impairments are not uncommon in iNPH, and our findings could suggest that declining memory is included in the early development of iNPH.

## Data Availability

All data are available upon request. Requests can be sent to the corresponding author Otto Lilja-Lund.
